# Differential biologic effects of CPD and 6-4PP UV-induced DNA damage on the induction of apoptosis and cell-cycle arrest

**DOI:** 10.1186/1471-2407-5-135

**Published:** 2005-10-19

**Authors:** Hsin-Lung Lo, Satoshi Nakajima, Lisa Ma, Barbara Walter, Akira Yasui, Douglas W Ethell, Laurie B Owen

**Affiliations:** 1 Division of Biomedical Sciences, University of California Riverside, Riverside, CA 92521 USA; 2 Department of Molecular Genetics, Institute of Development, Aging and Cancer, Tohoku University, Sendai, 980-8575 Japan; 3 BioLegend, Inc., 8395 Camino Santa Fe, Suite E, San Diego, CA 92121 USA

## Abstract

**Background:**

UV-induced damage can induce apoptosis or trigger DNA repair mechanisms. Minor DNA damage is thought to halt the cell cycle to allow effective repair, while more severe damage can induce an apoptotic program. Of the two major types of UV-induced DNA lesions, it has been reported that repair of CPD, but not 6-4PP, abrogates mutation. To address whether the two major forms of UV-induced DNA damage, can induce differential biological effects, NER-deficient cells containing either CPD photolyase or 6-4 PP photolyase were exposed to UV and examined for alterations in cell cycle and apoptosis. In addition, pTpT, a molecular mimic of CPD was tested *in vitro *and *in vivo *for the ability to induce cell death and cell cycle alterations.

**Methods:**

NER-deficient XPA cells were stably transfected with CPD-photolyase or 6-4PP photolyase to specifically repair only CPD or only 6-4PP. After 300 J/m^2 ^UVB exposure photoreactivation light (PR, UVA 60 kJ/m^2^) was provided for photolyase activation and DNA repair. Apoptosis was monitored 24 hours later by flow cytometric analysis of DNA content, using sub-G1 staining to indicate apoptotic cells. To confirm the effects observed with CPD lesions, the molecular mimic of CPD, pTpT, was also tested *in vitro *and *in vivo *for its effect on cell cycle and apoptosis.

**Results:**

The specific repair of 6-4PP lesions after UVB exposure resulted in a dramatic reduction in apoptosis. These findings suggested that 6-4PP lesions may be the primary inducer of UVB-induced apoptosis. Repair of CPD lesions (despite their relative abundance in the UV-damaged cell) had little effect on the induction of apoptosis. Supporting these findings, the molecular mimic of CPD, (dinucleotide pTpT) could mimic the effects of UVB on cell cycle arrest, but were ineffective to induce apoptosis.

**Conclusion:**

The primary response of the cell to UV-induced 6-4PP lesions is to trigger an apoptotic program whereas the response of the cell to CPD lesions appears to principally involve cell cycle arrest. These findings suggest that CPD and 6-4 PP may induce differential biological effects in the UV-damaged cell.

## Background

More than one million cases of skin cancer are diagnosed in the U.S. annually, resulting in 9,600 deaths, 7,400 of which are from metastatic melanoma [1]. Absorption of ultraviolet (UV) light produces two predominant types of DNA damage, cyclobutane pyrimidine dimers (CPD) [2] and pyrimidine (6-4) pyrimidone photoproducts (6-4PP) [3]. The result is a transition of C to T and CC to TT [4], which are the most frequent mutations of *p53 *in both human and mouse skin cancers [5,6]. UV damaged DNA causes torsional strain and is usually repaired by nucleotide excision repair (NER) or base excision repair (BER). After UV exposure, cells activate p53 and stall the cell cycle for repair [7,8]. If the damage is too severe, the cell will trigger apoptosis to get rid of a DNA damaged, potentially mutant cell [9]. But how does the cell determine when UV-damaged DNA can be repaired or when apoptosis should be initiated?

Previous studies have reported that CPD, but not 6-4PP, lesions are the major source of lasting UV-induced mutations [10], as CPD is usually at a 5 to 10 fold higher frequency than 6-4PP [11,12]. The repair of CPD lesions by exogenous CPD photolyase has been shown to reduce apoptosis both *in vivo *[13] and *in vitro *[14], but such studies did not examine the effect of 6-4PP lesions. Indeed, most previous studies have assumed that the amount of DNA damage is the deciding factor in a cell's fate with both lesions weighing in equally for biologic effect.

In this study, we have investigated whether UV-induced CPD and 6-4PP DNA damage have differential effects on the induction of apoptosis and cell-cycle arrest. To control DNA repair in UV-exposed human cells we have used photolyases, the DNA repair enzymes that are employed widely in the animal kingdom, but not in man [15]. Bacteria, fungi, plants, invertebrates, and many vertebrates, but not humans, use photolyases to repair UV-induced DNA damage [15,16]. This light-dependent (360–420 nm, visible~UVA) photolyase mediated repair of CPD or 6-4PP is referred to as photoreactivation (PR). By expressing photolyases in NER-defective cells we have been able to individually analyze the impact of residual CPD and 6-4PP lesions on apoptosis and cell cycle. Our findings demonstrate that the "type" of DNA lesion is as important as the total "amount" of damaged DNA in the balance of life and death, as it relates to apoptosis after UVB exposure.

## Materials and methods

### Chemicals and Cell Culture

All chemicals were purchased from Sigma (St. Louis, MO) unless otherwise noted. The SV40-transformed human cell line derived from an XPA patient, XP12ROSV, was stably transfected with expression vectors containing cDNAs for either 6-4PP photolyase, CPD photolyase, both types of photolyases, or empty vector controls as previously described [10,17]. Cells were maintained in Eagle's minimum essential medium (MEM) containing 10% heat-inactivated fetal bovine serum, 2 mM L-glutamine, 1 mM sodium pyruvate, 100 units/ml penicillin, 100 μg/ml streptomycin, 1× MEM vitamins solution, and 1× MEM non-essential amino acids at 37°C in a humidified 5% CO_2 _incubator.

### UV-irradiation and photoreactivation

Twenty-four hours prior to UV-irradiation, media was changed to phenol-red free culture medium to prevent the absorption of energy by phenol red. The UV source used was a Spectrolinker XL-1000 (Spectronics Corp., Westbury, NY) containing five 8-watt UV tubes. Two different UV wavelength tube sets were used, UVA 365 nm, and UVB 312 nm. For induction of DNA damage and apoptosis, cells were irradiated at 300 J/m^2 ^UVB in 100 mm cell culture dishes without lids. After irradiation, cells were treated with UVA, which serves as photo-reactivation energy for photolyase activity (30 min, 60 kJ/m^2^). Following treatments, cells were incubated for 24 h prior to harvesting. Cells were harvested by washing with pre-warmed PBS and adding trypsin for 2 min. Cell pellets were collected after centrifugation at 1000 × *g *for 5 min.

### Radio-immunoassay (RIA) of 6-4PP and CPD

Genomic DNA from UV exposed cells was isolated with a Wizard Genomic DNA purification kit (Promega, Madison, WI) 12 hours after 300 J/m^2 ^UVB irradiation. UV-induced DNA damage was detected using specific antibodies and a radioimmunoassay described previously [18].

### DNA damage detection by Immuno-Dot-Blot

The repair of CPD and 6-4PP lesions after photoreactivation was measured by an Immuno-Dot-Blot assay using the CPD-specific monoclonal antibody and the 6-4PP-specific monoclonal antibody (Kamiya Biomedical, WA). After UV treatment and PR, cellular DNA was isolated and then denatured in TE buffer (10 mM Tris-CL and 1 mM EDTA, pH 7.5) by boiling for 5 min. Thesamples were dot-blotted onto a Hybond N+ membrane (Amersham, NJ) using 200 ng of DNA for the CPD assay and 1 μg of DNA for the 6-4PP assay. The membrane was dried by baking on a 80°C plate for 1 hour then DNA was fixed to the membrane for 20 min by soaking in 0.4 N NaOH on a filter paper. The membranes were blocked overnight in phosphate-buffered saline, 0.2% Tween 20 (PBS-T) containing 5% (w/v) skim milk. After washing in PBS-T, the membranes were incubated for 2 h at room temperature with anti 6-4PP antibody and anti CPD monoclonal antibody. After washing, membranes were incubated for 1 h with peroxidase conjugated goat anti-mouse antibody (Jackson ImmunoResearch, PA). Signals were detected with a chemiluminescence kit (Amersham, NJ).

### Western blot

Whole cell lysates were prepared from pelleted cells in lysis buffer [20 mM Tris-HCl (pH 8.0), 150 mM sodium chloride, 10% glycerol, 1% Triton-X 100, 2 mM sodium orthovanadate, 2 mM EDTA and 1× complete protease inhibitor cocktail (Roche, Germany)]. Total protein concentrations in lysate preparations were determined using a BCA assay and 15 μg total protein was loaded in each lane of a 10% SDS-PAGE gel. Proteins were resolved by electrophoresis and blotted onto nitrocellulose membranes (Hybond ECL, Amersham Pharmacia Biotech, Buckinghamshire, United Kingdom). Blots were blocked with 5% milk in PBS for 1 h prior to incubation with primary antibodies. To confirm photolyase expression, blots were probed with rabbit antibodies specific for 6-4PP photolyase or CPD photolyase (Dr. Yasui, Tohoku University, Sendai, Japan). The blots were washed 3× with PBST (0.5% Tween 20) and incubated with secondary peroxidase-conjugated goat anti-rabbit antibodies for 1 h (Jackson ImmunoResearch Laboratories, Inc., West Grove, PA). Blots were rinsed 3× with PBST and antibody binding was detected with ECL (Amersham Pharmacia Biotech).

### Flow cytometric measurement of cell cycle and apoptosis

Following UV or pTpT treatment, cells were harvested and fixed in 70% ethanol at -20°C overnight. Cells were then pelleted and resuspended in 125 μl of PBS containing 0.2% Triton X-100 solution and 12 Kunitz units of ribonuclease A (Sigma) for 15 min at room temperature, followed by the addition of 130 μl propidium iodide (PI) at 0.1 mg/ml. Cells were maintained in the dark and analyzed by flow cytometry using a Becton Dickinson FACScan (San Jose, CA). After excluding cell debris and doublets, DNA content and cell cycle were analyzed using Cellquest-Pro software. Cells showing DNA content less than the peak for G_1/_G_0 _were considered sub-diploid and apoptotic.

### Mimic of damaged DNA by pTpT

Thymidine dinucleotides, pTpT, (Midland Certified Reagent, Midland, TX) were used to mimic UV-induced DNA damage. *In vitro*, 100 μM pTpT was added to Jurkat cell culture medium, the same concentration of adenosine dinucleotides (pApA) was used for a negative control. Cells were harvested 1 h or 24 h after treatment to examine cell cycle. For *in vivo *studies, pTpT and pApA were dissolved in propylene glycol and 100 μl was applied to the shaved backs of C57BL/6 mice to cover an area at 200 μM per 2 cm^2 ^(Jackson Labs, Bar Harbor, ME). All mouse studies were performed in accordance with NIH and Institutional Animal Care and Use Committee guidelines. The skin was shaved 24 hours before treatment. Mice were exposed to a single dose of 4 kJ/m^2 ^UVB as positive controls. Mice were sacrificed 24 hours after treatment, with 3 mice for each treatment and 3 independent experiments performed. Formaldehyde-fixed skin biopsies were paraffin embedded. Seven-micron sections were made with a microtome and mounted onto charged glass slides. Apoptosis was assessed by staining for DNA fragmentation using the DeadEnd Fluorometric TUNEL System (Promega, Madison, WI). All TUNEL-positive cells in the epidermis of each section were counted using a fluorescence microscope. Each counted section was measured for length using calipers and using an apoptotic index generated to show the number of TUNEL-positive cells per unit of length.

## Results and discussion

### CPD is the dominant DNA lesion in NER-deficient cells after UVB irradiation

**Figure 1:**
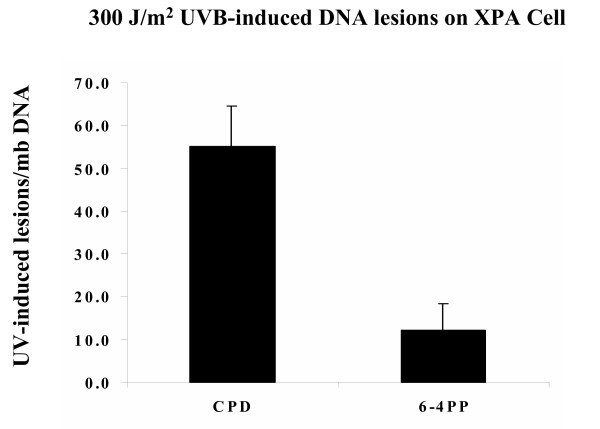
**Radio-immunoassay (RIA) of UV-induced 6-4PP and CPD**. Twelve hours after UVB irradiation, genomic DNA was isolated from treated cells and assayed for UV-induced DNA damage using lesion-specific antibodies. An average of fifty-five CPD and twelve 6-4PP lesions per million bases were detected after exposure to 300 J/m^2 ^UVB. Results are the average of duplicate assays from two independent experiments. Error bars show standard deviation.

XPA cells mutated in the *xpa *gene cannot perform nucleotide excision repair (NER) and therefore accumulate large amounts of UV-induced DNA damage after UVB exposure. XPA cells were irradiated with 300 J/m^2 ^UVB and assayed for UV-induced DNA damage using mutation-specific radioimmunoassays (RIA). The 300 J/m^2 ^UVB dose was chosen as there was little acute, UV-induced oxidative cell death induced under these conditions, while there was sufficient levels of DNA damage to reproducibly measure decreases following photolyase repair (doses between 50~1500 J/m^2 ^were screened initially). Because 6-4PP lesions occur at a lower frequency than CPD lesion, the 300 J/m^2 ^dose was chosen as the lowest dose of UVB that produced high enough numbers of 6-4PP lesions such that repair could be accurately measured following photolyase treatment (>10 lesions per 10^6 ^DNA pairs). At this UVB dose, a substantial number of CPD lesions were observed (averaging 55 CPD per 10^6 ^DNA pairs) and fewer, but reproducible number of 6-4PP lesions (averaging at 12 6-4PP per 10^6 ^DNA pairs). The average number of lesions in unrepaired cells in two independent experiments is shown in Figure 1. Our results show that CPD are the predominant UV-induced DNA lesions and are approximately five-fold more prevalent than 6-4PP. These results are consistent with other studies that show 6-4PP lesions occur at 15–30% the frequency of CPD lesion following UV damage [12].

### Repair of 6-4PP significantly reduces apoptosis

**Figure 2:**
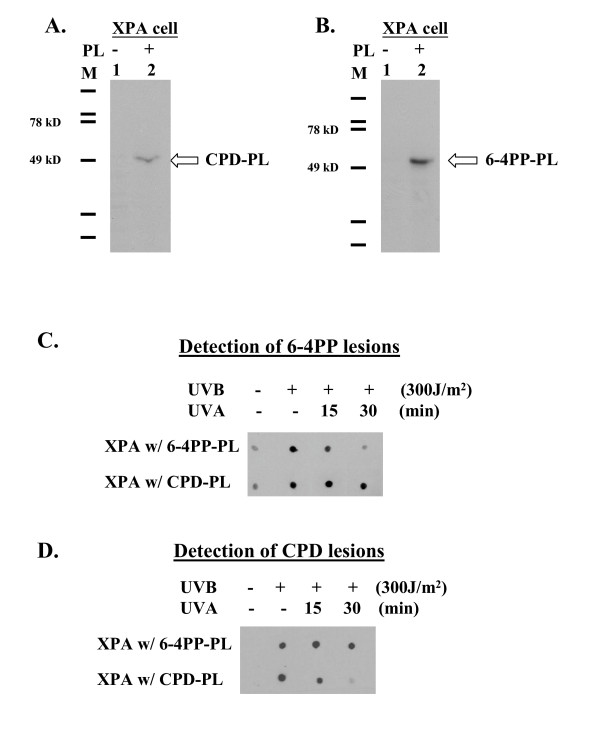
**Detection of photolyase expression and function**. Photolyase expression was confirmed by western blots of whole cell lysates from stably transfected XPA cell lines containing empty vector or both photolyases. Blots were probed with antibodies specific for CPD-PL (A) and 6-4PP-PL (B). Photolyase repair of UV induced DNA damage in XPA cells was detected by immuno-dot-blot, using the same antibodies. Representative examples of 6-4PP (C) and CPD (D) lesion studies are shown.

**Figure 3:**
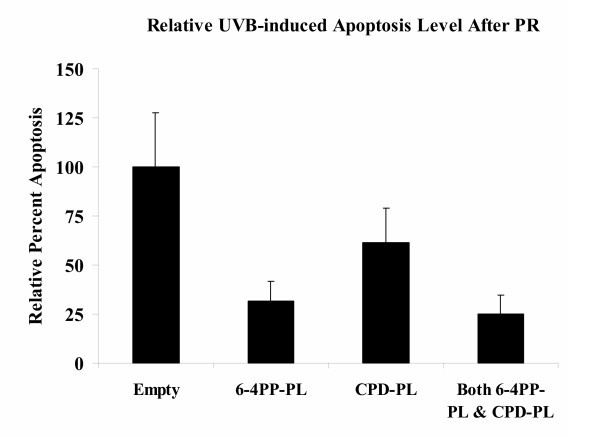
**Impact of photoreactivation on UVB-induced apoptosis**. Apoptosis in XPA cells was monitored at a 24 hour time point following exposure to 300 J/m^2 ^UV and photoreactivation-induced repair. Apoptosis was examined by PI staining and FACS, gating the sub-G1 population as apoptotic. Apoptotic values were normalized to empty vector containing cells (amount of apoptosis set as 100%). Bar graphs show the relative amount of apoptosis for control cells, cells expressing CPD-PL alone (40% rescue), cells expressing 6-4PP-PL alone (70% rescue), or cell expressing both-PLs (75% rescue).

We examined the effects of repairing either CPD or 6-4PP lesions by stably expressing photolyase cDNAs in XPA cells. Several studies have shown that these photolyase enzymes can efficiently and specifically repair DNA lesions within one hour when provided with photo-reactivation light [10,14,19]. Stable expression and function of each photolyase in XPA cells was confirmed by western blot and immuno-dot blot (Fig 2). XPA cells expressing 6-4PP-PL only repair 6-4PP type lesions, vice versa, CPD-PL only repair CPD type lesions (Fig 2C and 2D). Using this system we were able to independently repair one kind of lesion and thereby examine the biological effects of the other in relative isolation. Background apoptosis of XPA cells, as measured by sub-G1 DNA, is 1–3% for non-irradiated cells. Twenty-four hours after exposure to UVB and PR (60 kJ/m^2 ^UVA), 20.9% of empty vector transfected cells were apoptotic. UV-exposed XPA cells that expressed both photolyases and received PR, showed only 5.3% apoptosis, a reduction of approximately 75%. Cells expressing only one of the photolyases showed considerably different responses depending upon which enzyme was expressed. Repair of 6-4PP lesions reduced apoptosis to 6.6%, while repair of CPD lesions only reduced apoptosis to 12.9%. Although CPD is the major type of UV-induced DNA lesion (82%, about five times more than 6-4PP) repair of CPD only reduces apoptosis by 40% (Fig 3). In contrast, 6-4PP lesions comprise only 18% of UVB-induced DNA lesions, but account for 70% of the apoptosis (Fig 3). Indeed, repairing 6-4PP lesions alone suppressed apoptosis as much as repairing both types of DNA lesions. Similar findings were observed when Annexin V was used to measure apoptotic responses; suggesting that both sub-diploid DNA and phosphatidylserine exposure on the outer membrane leaflet are impacted by photolyase repair (data not shown). These findings indicate that 6-4PP lesions are more potent inducers of apoptosis than CPD lesions.

### The CPD mimic, pTpT, stalls the cell cycle but does not significantly induce apoptosis

UV exposure induces mainly CPD lesions (Fig. 1) that are typically repaired by NER. Thymidine dinucleotides, pTpT a mimic of CPD, are structurally similar to the small DNA fragments released during NER and have been reported to mimic UV-damaged DNA by activating p53 and proliferating cell nuclear antigen (PCNA) to enhance DNA repair [20]. These dinucleotides have also been reported to mimic damaged DNA and to increase melanogenesis [21] in normal human melanocytes and in human melanoma cells. The monomeric form of thymidine, pT, [22] or pApA [21] does not increase melanogenesis indicating the effect of pTpT in mimicking CPD lesions.

**Figure 4:**
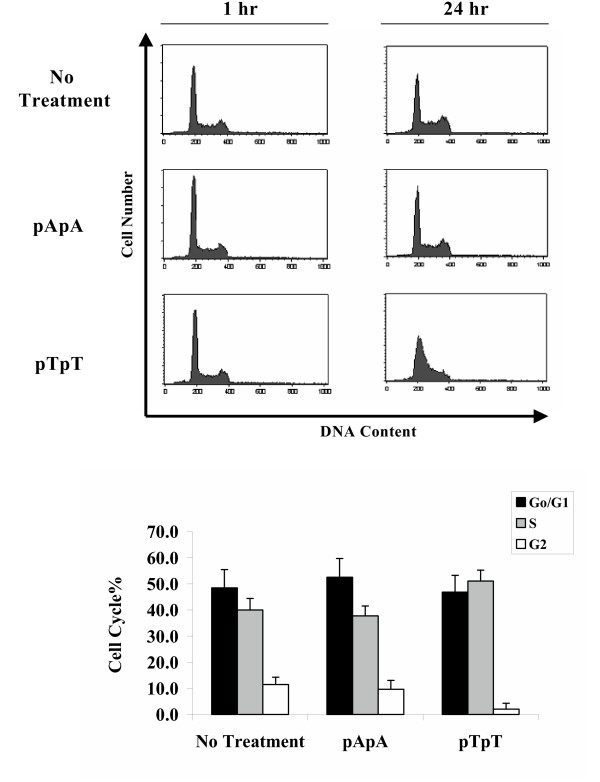
**Effects of pTpT on cell cycle and apoptosis *in vitro***. **A**. Jurkat cells were exposed to pTpT (100 μM) or pApA (100 μM) *in vitro *and cell cycle analysis performed after 1 and 24 hours by flow cytometry as described in Materials and Methods. Results show representative histograms from five independent experiments. Note that only pTpT stalls the cell cycle, not pApA, and no increase in apoptotic sub-G1 DNA content was observed with either pTpT or pApA. **B**. Bar graph showing percentage of cells in each phase of the cell cycle in cells treated with pTpT or pApA for 24 hrs.

UV can upregulate the death receptor apoptotic pathway to eradicate DNA damaged cells [23,24]. Lymphocytes are especially sensitive to DNA damage-induced apoptosis. Jurkat cells, a human T-cell line, provides a well-established model for death receptor (Fas-FasL) mediated apoptosis in response to either UV and/or chemotherapeutic drugs [25]. We examined if pTpT could induce Fas-FasL mediated apoptosis *in vitro *and *in vivo*. The addition of pTpT to Jurkat cells stalled the cell cycle, but did not induce FasL (data not shown) or apoptosis (as seen by sub-diploid peak analysis, Fig. 4A). Jurkat cells exposed to the same concentration of control oligonucleotide, pApA, did not show any cell cycle disruption or increased apoptosis. The relative percentage cells in each phase of the cell cycle for pTpT- and pApA-treated cells is shown in Fig. 4B. Note the increased proportion of cells in S-phase and decreased proportion of cells in G2/M phase for pTpT treated cells as compared to controls and pApA treated cells.

**Figure 5:**
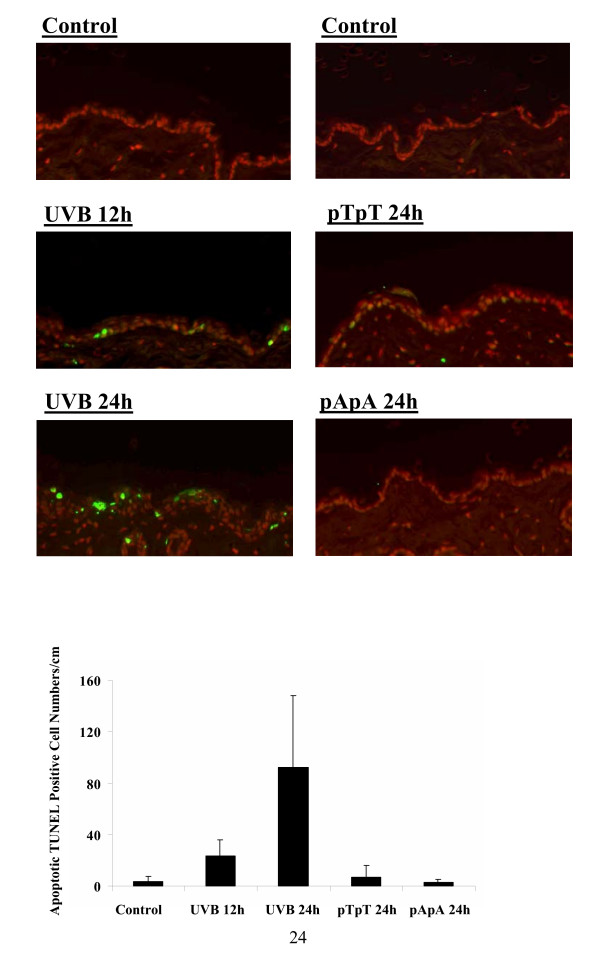
**CPD mimic, pTpT, does not induce apoptosis *in vivo***. TUNEL-stained sections from mouse dorsal skin coated with pTpT or pApA, or exposed to a single dose 4 kJ/m^2 ^UVB as a positive control. (A) Green fluorescence indicates 3' DNA breaks, representative of apoptotic cells. (B) Numbers of TUNEL positive cells in skin sections from three separate experiments, consisting of three mice in each experimental group. Apoptotic values indicate the number of TUNEL-positive cells by tissue length. Pictures were taken on a Nikon ECLIPSE TE2000-U microscope under FITC illumination at 400×.

We also tested whether pTpT could alter FasL-mediated apoptosis in an established *in vivo *model. For these experiments, solvent containing 200 μM pTpT or 200 μM pApA was applied on the shaved dorsal skin of C57BL/6 mice. Mice exposed to a single dose of 4 kJ/m^2 ^of UVB were used as positive controls. Apoptosis was assessed on paraffin embedded formaldehyde-fixed skin biopsies using the DeadEnd Fluorometric TUNEL System (Promega). Although UV-induced apoptosis was observed 12 hours after UV treatment and continued to increase at least until 24 hours following UV exposure (Fig 5A), pTpT did not induce apoptosis *in vivo *in concert with our findings *in vitro *(Fig. 5B). Taken together, our findings suggest that pTpT, the mimic of CPD, is not a potent inducer of apoptosis, but inhibits cell cycle progression. Similar results have been observed with human melanocytes *in vitro*, where pTpT has been shown to induces melanogenesis and stall the cell cycle in the absence of increased apoptosis [22].

Our finding that repair of 6-4 PP lesions was more effective in the blockade of apoptosis than the repair of CPD lesions would seem to be contradictory to published findings that transgenic mice expressing the CPD photolyase in the skin showed dramatically reduced levels of UV-induced apoptosis, erythema, and hyperplasia [13]. One underlying factor that may contribute to the effects of CPD on apoptosis induction is the p53 status of the cell. Because we [9] have shown that p53 can induce upregulation of death receptors such as Fas which plays a key role in UV-induced apoptosis *in vivo * [26], it is imperative to consider the p53 status of the cells/animals used for study. In the case of the XP cells used for the current studies, p53 is mutant (data not shown). When p53 is wild-type, CPD lesions may be more robust in the induction of apoptosis. Other studies have shown that transgenic animals expressing the CPD photolyase show superior resistance to sunlight-induced carcinogenesis [27]. These findings may relate to the fact that UV-induced apoptosis is decreased in the skin of chronically irradiated mice consistent with the induction p53 mutations [23]. Alternately stated, when p53 is wild-type CPD lesions may be a more powerful inducer of apoptosis, however, once p53 becomes mutated (as is commonly observed for non-melanoma skin cancers) CPD lesions may act to induce cell cycle arrest. It will be especially interesting to examine transcription coupled repair efficiency following photolyase treatments in wild-type and mutant p53 containing cells as reports have indicated that transcription coupled repair efficiency can determine cell cycle progression and apoptosis in UV-irradiated cells [28]. Future studies will address the impact of p53 status on the biological effects of CPD and 6-4 PP lesion repair as well as the effects of such repair on transcription coupled repair and global genome repair.

## Conclusion

The UVB-induced DNA lesions, CPD and 6-4PP, show differential biological effects with respect to the induction of apoptosis and cell cycle arrest. CPD lesions are the most abundant following UVB exposure and photolyase-induced repair of these lesions decreased the overall apoptotic response by approximately 40% compared with cells receiving no PR repair. Repair of 6-4PP lesions which account for approximately 20% of the total UV-induced DNA damage, on the other hand, decreased the overall apoptotic response by approximately 75% (almost equivalent to that using both CPD and 6-4PP photolyases together). These findings suggest that the 6-4 PP lesion is more potent in the induction of UV-induced apoptosis. In contrast, the CPD lesion appears to be more potent in the induction of cell cycle arrest. Using the CPD mimic, pTpT, we documented that cell cycle arrest occurred *in vitro *after treatment in the absence of increased apoptosis. Similar findings were also observed in vivo using the pTpT molecular mimic of CPD lesions. Therefore CPD lesions will halt the cell cycle to effect repair that may or may not eliminate the damage, thus increasing the relative probability that these kinds of lesions will accumulate leading to mutation. In support of this premise, You et al. [10] have documented that CPD lesions account for the majority of UV-induced mutations in mammalian cells. The more potent apoptosis-inducing activity of 6-4PP lesions may induce apoptosis and "erase" the damaged cell, thus decreasing its carcinogenic potential.

## Authors' contributions

HL and LOS initiated the research design, execution, coordination and writing. SN and AY carried out the XPA stable transfection cell line production. BW performed the FAScan assay. LM participated in the pTpT mimics CPD study. DE participated in study design and manuscript editing. All the authors have read and approved the final manuscript.

## Pre-publication history

The pre-publication history for this paper can be accessed here:



## References

[B1] American Cancer Society. [http://wwwcancerorg/docroot/PED/content/ped_7_1_What_You_Need_To_Know_About_Skin_Cancerasp?sitearea=PED].

[B2] Lippke JA, Gordon LK, Brash DE, Haseltine WA (1981). Distribution of UV light-induced damage in a defined sequence of human DNA: detection of alkaline-sensitive lesions at pyrimidine nucleoside-cytidine sequences. Proc Natl Acad Sci U S A.

[B3] Mitchell DL, Nairn RS (1989). The biology of the (6-4) photoproduct. Photochem Photobiol.

[B4] Ananthaswamy HN, Loughlin SM, Cox P, Evans RL, Ullrich SE, Kripke ML (1997). Sunlight and skin cancer: inhibition of p53 mutations in UV-irradiated mouse skin by sunscreens. Nat Med.

[B5] Soehnge H, Ouhtit A, Ananthaswamy ON (1997). Mechanisms of induction of skin cancer by UV radiation. Front Biosci.

[B6] Ananthaswamy HN, Bowden GTFSM (1997). Ultraviolet light as a carcinogen.. Chemical Carcinogens and Anticarcinogens.

[B7] el-Deiry WS, Tokino T, Velculescu VE, Levy DB, Parsons R, Trent JM, Lin D, Mercer WE, Kinzler KW, Vogelstein B (1993). WAF1, a potential mediator of p53 tumor suppression. Cell.

[B8] Hermeking H, Lengauer C, Polyak K, He TC, Zhang L, Thiagalingam S, Kinzler KW, Vogelstein B (1997). 14-3-3 sigma is a p53-regulated inhibitor of G2/M progression. Mol Cell.

[B9] Hill LL, Ouhtit A, Loughlin SM, Kripke ML, Ananthaswamy HN, Owen-Schaub LB (1999). Fas ligand: a sensor for DNA damage critical in skin cancer etiology. Science.

[B10] You YH, Lee DH, Yoon JH, Nakajima S, Yasui A, Pfeifer GP (2001). Cyclobutane pyrimidine dimers are responsible for the vast majority of mutations induced by UVB irradiation in mammalian cells. J Biol Chem.

[B11] Meador JA, Walter RB, Mitchell DL (2000). Induction, distribution and repair of UV photodamage in the platyfish, Xiphophorus signum. Photochem Photobiol.

[B12] Bissonauth V, Drouin R, Mitchell DL, Rhainds M, Claveau J, Rouabhia M (2000). The efficacy of a broad-spectrum sunscreen to protect engineered human skin from tissue and DNA damage induced by solar ultraviolet exposure. Clin Cancer Res.

[B13] Schul W, Jans J, Rijksen YM, Klemann KH, Eker AP, de Wit J, Nikaido O, Nakajima S, Yasui A, Hoeijmakers JH, van der Horst GT (2002). Enhanced repair of cyclobutane pyrimidine dimers and improved UV resistance in photolyase transgenic mice. Embo J.

[B14] Chigancas V, Miyaji EN, Muotri AR, de Fatima Jacysyn J, Amarante-Mendes GP, Yasui A, Menck CF (2000). Photorepair prevents ultraviolet-induced apoptosis in human cells expressing the marsupial photolyase gene. Cancer Res.

[B15] Yasui A, McCready SJ (1998). Alternative repair pathways for UV-induced DNA damage. Bioessays.

[B16] Yasui AAPME, Hoekstra JANMF (1998). DNA Photolyase. DNA Damage and Repair.

[B17] Nakajima S, Lan L, Kanno S, Takao M, Yamamoto K, Eker AP, Yasui A (2004). UV light-induced DNA damage and tolerance for the survival of nucleotide excision repair-deficient human cells. J Biol Chem.

[B18] Mitchell DL, Byrom M, Chiarello S, Lowery MG (2001). Effects of chronic exposure to ultraviolet B radiation on DNA repair in the dermis and epidermis of the hairless mouse. J Invest Dermatol.

[B19] Chigancas V, Batista LF, Brumatti G, Amarante-Mendes GP, Yasui A, Menck CF (2002). Photorepair of RNA polymerase arrest and apoptosis after ultraviolet irradiation in normal and XPB deficient rodent cells. Cell Death Differ.

[B20] Maeda T, Eller MS, Hedayati M, Grossman L, Gilchrest BA (1999). Enhanced repair of benzo(a)pyrene-induced DNA damage in human cells treated with thymidine dinucleotides. Mutat Res.

[B21] Eller MS, Gilchrest BA (2000). Tanning as part of the eukaryotic SOS response. Pigment Cell Res.

[B22] Pedeux R, Al-Irani N, Marteau C, Pellicier F, Branche R, Ozturk M, Franchi J, Dore JF (1998). Thymidine dinucleotides induce S phase cell cycle arrest in addition to increased melanogenesis in human melanocytes. J Invest Dermatol.

[B23] Ouhtit A, Gorny A, Muller HK, Hill LL, Owen-Schaub L, Ananthaswamy HN (2000). Loss of Fas-ligand expression in mouse keratinocytes during UV carcinogenesis. Am J Pathol.

[B24] Hill LL, Shreedhar VK, Kripke ML, Owen-Schaub LB (1999). A critical role for Fas ligand in the active suppression of systemic immune responses by ultraviolet radiation. J Exp Med.

[B25] Kasibhatla S, Brunner T, Genestier L, Echeverri F, Mahboubi A, Green DR (1998). DNA damaging agents induce expression of Fas ligand and subsequent apoptosis in T lymphocytes via the activation of NF-kappa B and AP-1. Mol Cell.

